# Methylated Trivalent Arsenic-Glutathione Complexes are More Stable than their Arsenite Analog

**DOI:** 10.1155/2008/539082

**Published:** 2008-05-15

**Authors:** Andrew J. Percy, Jürgen Gailer

**Affiliations:** ^1^Department of Chemistry, University of Calgary, 2500 University Drive N.W., Calgary, Alberta, Canada T2N 1N4

## Abstract

The trivalent arsenic glutathione complexes arsenic triglutathione, methylarsonous diglutathione, and dimethylarsinous glutathione are key intermediates in the mammalian metabolism of arsenite and possibly represent the arsenic species that are transported from the liver to the kidney for urinary excretion. Despite this, the comparative stability of the arsenic-sulfur bonds in these complexes has not been investigated under physiological conditions resembling hepatocyte cytosol. Using size-exclusion chromatography and a glutathione-containing phosphate buffered saline mobile phase (5 or 10 mM glutathione, pH 7.4) in conjunction with an arsenic-specific detector, we chromatographed arsenite, monomethylarsonous acid, and dimethylarsinous acid. The on-column formation of the corresponding arsenic-glutathione complexes between 4 and 37°C revealed that methylated arsenic-glutathione complexes are more stable than arsenic triglutathione. The relevance of these results with regard to the metabolic fate of arsenite in mammals is discussed.

## 1. INTRODUCTION

The metalloid arsenic (As) ranks 53rd amongst elements in the Earth's crust [1], where it is predominantly found in sulfidic ores, such as arsenopyrite (FeAsS) and orpiment (As_2_S_3_) [2]. Natural and anthropogenic activities, however, mobilize geogenic arsenic into the aquatic environment, including drinking water, where arsenite (As(III)) and/or arsenate (As(V)) are the most prevalent oxyanions [2]. Because the chronic ingestion of only 50–200 *μ*g/day of inorganic arsenic is associated with cancers of the skin, the liver, the lungs, the kidneys, and the bladder in humans [3–7], the exposure of humans to concentrations of inorganic As in drinking water that are unsafe for human consumption currently affects ∼100 million people [8]. In fact, the low-level As poisoning tragedy that is currently unfolding in parts of India and Bangladesh has been referred to as the largest mass poisoning in history [9, 10].

**Figure 1:**
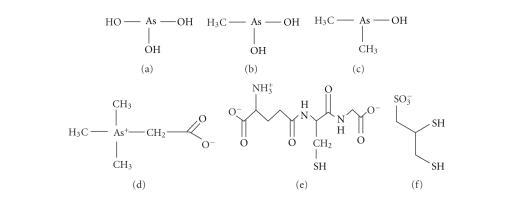
Solution species at pH 7.4 of (a) arsenite, As(III), (b) monomethylarsonous acid, MMA(III), (c) dimethylarsinous acid, DMA(III), (d) arsenobetaine, AsB, (e) glutathione, GSH, and (f) sodium 2,3-dimercapto-1-propanesulfonate, DMPS [18, 19].

Investigations carried out in the 1970s, which aimed to identify As-containing metabolites in human urine,indicated two pentavalent organoarsenicals monomethylarsonic acid (MMA(V)) and dimethylarsinic acid (DMA(V)), in addition to As(III) and As(V) [11]. More recently, two additional trivalent organoarsenicals—monomethylar-sonousacid [MMA(III), CH_3_As(OH)_2_, Figure 1(b), [12–16]] and dimethylarsinous acid [DMA(III), (CH_3_)_2_AsOH, Figure 1(c), [12, 14, 15]]—have also been identified (we note that other As-containing metabolites, such as thiodimethylarsenopropanoic acid and thiodimethylarsenobutanoic acid, have been recently identified in human urine as metabolites of ingested arsenolipids [17]).

Studies into the mammalian metabolism of As(III) (Figure 1(a)) have revealed a high propensity of this species to react with soft ligands, such as the thiol group of cysteine [20–24]. Because the cysteine-containing tripeptide glutathione (GSH, Figure 1(e)) is the most abundant endogenous thiol in mammalian hepatocyte cytoplasm (5 mM [25], whereas the concentration of L-cysteine is 0.2–0.5 mM [26]), the chemical reaction of As(III) with three successive mole equivalents of GSH to arsenic triglutathione [As(SG)_3_], according to (1), is possibly the first step in the hepatic metabolism of this oxyanion *in vivo.*
(1)
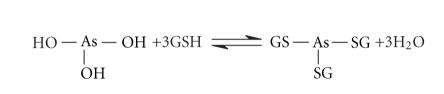

Given that As(III) is known to be enzymatically methylated in the liver of most mammals [27–30], the detection of methylarsonous diglutathione [CH_3_As(SG)_2_] in bile of As(III) treated rats [31–33] indicates that this metabolite is formed in the liver, according to (2): 
(2)
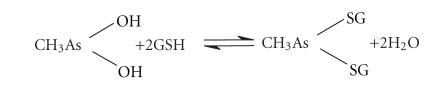

In view of the fact that DMA(III) has also been identified in mammalian urine [12, 14, 15], it is chemically feasible that dimethylarsinous glutathione [(CH_3_)_2_AsSG] is also formed in the liver, according to (3):

(3) $$(\text{C}{\text{H}}_{3}{)}_{2}\text{A}\text{s}\text{-O}\text{H}+\text{G}\text{S}\text{H}⇌(\text{C}{\text{H}}_{3}{)}_{2}\text{A}\text{s}\text{-S}\text{G}+{\text{H}}_{2}\text{O}.$$

With respect to the biomethylation mechanism of As(III) in mammals, there are currently two proposed pathways [34, 35]. The first one involves the enzymatic oxidative methylation of As(III) to MMA(V), which is enzymatically reduced to MMA(III), and can then undergo a second enzymatic oxidative methylation reaction to DMA(V) [29, 36–38]. The alternative, more recently proposed scheme involves the stepwise enzyme-mediated reductive methylation of As(SG)_3_ to CH_3_As(SG)_2_ and (CH_3_)_2_AsSG, using the same methyl donor as in scheme one—S-adenosyl-L-methionine [39, 40]. In this latter scheme, CH_3_As(SG)_2_ is then oxidized to MMA(V) by endogenously generated H_2_O_2_ while DMA(V) is produced in a similar manner from (CH_3_)_2_AsSG. The entire biomethylation mechanism in hepatocytes, however, is not completely understood because the concerted sequence of binding events of As(III) to endogenous small-molecular-mass thiols [41] and the methylating enzymes remains unknown [34].

Irrespective of the mechanism of biomethylation, As(SG)_3_, CH_3_As(SG)_2_, and (CH_3_)_2_AsSG likely play important roles in the transport of methylated arsenicals from the liver to the bloodstream [42–46]. Despite this, not much is known about the stability of the As-S bonds in CH_3_As(SG)_2_ and (CH_3_)_2_AsSG under conditions that resemble hepatocyte cytosol (phosphate buffered saline, pH 7.4, 5.0 mM GSH, 37°C). Previously, the formation of As(SG)_3_ from As(OH)_3_ and GSH has been studied under simulated physiological conditions (phosphate buffered saline, pH 7.4) by size-exclusion chromatography (SEC) [22]. This investigation revealed that the on-column formation of the As(SG)_3_ complex strongly depends on the GSH concentration in the mobile phase (5–7.5 mM favored) and preferably occurs at pH 6.0–8.0. An increase of the column temperature from 4 to 37°C (at constant GSH concentration and at a mobile phase pH of 7.4) resulted in retention shifts of the As(III) peaks toward the small-molecular-mass region, which indicated that the As-S bonds in As(SG)_3_ are rather labile. This finding is consistent with results attained in other studies [20, 47–49].

The mammalian metabolism of As(III) in hepatocytes is likely driven by its concerted interactions with cytosolic GSH (5.0 mM) and proteins. In order to gain insight into the role that GSH plays in the potential efflux of the generated As(III) metabolites from the liver into the systemic circulation, we have chromatographed As(III), MMA(III), and DMA(III) under conditions that resemble the chemical conditions of mammalian hepatocyte cytosol (phosphate buffered saline, 5–10 mM GSH, pH 7.4) in the absence of proteins. Investigations into the temperature-dependent retention behavior of As(III), MMA(III), and DMA(III) between 4 and 37°C provided insight into the formation (at 4°C) and the comparative stability (between 4 and 37°C) of the As-S bonds in the on-column formed complexes. Our results constitute a first step toward better understanding the disposal of As(III) metabolites from the liver to the bloodstream.

## 2. EXPERIMENTAL

**Caution**

*Since inorganic and organic arsenicals are established cytotoxins, genotoxins, and carcinogens [4, 50], measures must be implemented to reduce dermal and inhalatory exposure. To this end, synthesis and solution preparation were conducted in a glove box whilst wearing nitrile gloves and a respiratory mask.*

### 2.1. Chemicals

Sodium hydroxide, phosphate buffered saline (PBS) tablets, GSH, sodium 2,3-dimercapto-1-propanesulfonate (DMPS), blue dextran, oxidized glutathione (GSSG), and glycine (all > 95%) were purchased from Sigma-Aldrich (St.Louis, MO, USA). Sephadex G-15 (120 *μ*m mean spherical particles of dextran cross-linked with epichlorohydrin, exclusion limit: molecular mass (MM) < 1500 Da) was purchased from GE Healthcare. Sodium arsenite (NaAsO_2_) was obtained from GFS Chemicals (> 99%). The source of MMA(III) and DMA(III) was the solid methyldiiodoarsine (CH_3_AsI_2_) and the liquid dimethyliodoarsine [(CH_3_)_2_AsI], which were synthesized according to previously established procedures [51, 52]. To verify the purity of these trivalent organoarsenicals, ^1^H NMR spectra were obtained for these compounds (chemical shifts: 1.28 ppm for MMA(III) and 1.53 ppm for DMA(III)) and the starting material (chemical shifts: 1.44 ppm for MMA(V) and 1.97 ppm for DMA(V)). The obtained chemical shifts were found to be akin to those previously reported for these compounds [48, 53]. Arsenobetaine bromide (AsB) was synthesized according to a published procedure and its purity was verified by its melting point (experimental: 225°C versus reported: 227°C) [54]. All solutions, including mobile phases, were prepared with water from a simplicity water purification system (resistivity 18.2 MΩ⋅cm, Millipore).

### 2.2. Solutions

To avoid oxidation of GSH in aqueous mobile phases, all GSH-containing solutions were prepared fresh prior to each chromatographic run and used within 4 hours. PBS-buffer was prepared by dissolving PBS tablets in the appropriate volume of water. After the dissolution of a monothiol (5 or 10 mM GSH) and/or a dithiol (1 mM DMPS) in 400 mL PBS, the pH of the mobile phase was adjusted to 7.4 with sodium hydroxide (4.0 M) using a VWR Symphony SB20 pH meter and filtered through a 0.45 *μ*m Nylon membrane (Alltech).

Aqueous solutions of As(III), MMA(III), DMA(III), and AsB were prepared by dissolving NaAsO_2_, CH_3_AsI_2_, (CH_3_)_2_AsI, and AsB in water to obtain a concentration of 10 *μ*g As in 20 *μ*L. It is generally accepted that MMA(III) and DMA(III) are the only species that are formed upon hydrolysis of CH_3_AsI_2_ and (CH_3_)_2_AsI in water [18, 53]. Even though all chromatograms were generated with the acidic solutions of MMA(III) and DMA(III) (hydroiodic acid is formed during the hydrolysis of CH_3_AsI_2_ and (CH_3_)_2_AsI in water), the retention times (*t_r_*) of MMA(III) and DMA(III) were reduced by only ∼ 20 seconds when neutralized solutions were injected. To mitigate oxidation to the pentavalent state, As(III) and MMA(III) solutions were prepared every 14 days while DMA(III) was prepared fresh every 2-3 days [55–57]. All solutions were stored in septum glass vials at 4°C.

### 2.3. Instrumentation

The liquid chromatographic (LC) system consisted of a Waters 510 high-performance LC isocratic dual-piston pump, a Rheodyne six-port injection valve (20 *μ*L sample loop), a glass thermostatable SEC column packed with Sephadex G-15 (31×1.0 cm, GE Healthcare), and a cellulose filter on the column head (1.0 cm × 1.0 mm, GE Healthcare). After thermostating the packed column at the desired temperature of 4, 25, or 37°C (NESLAB RTE-7 digital one refrigerated bath, Thermo Scientific), it was equilibrated with at least 60 mL of mobile phase before the As compounds were injected. A flow rate of 1.0 mL/min was used throughout this study and each retention time was determined in triplicate (RSD <** **1.0%) after the individual injection of each arsenical.

**Figure 2:**
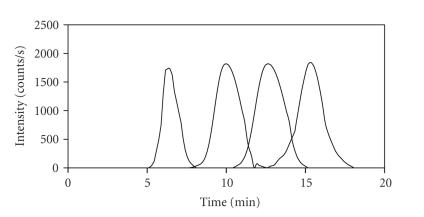
Superimposed chromatograms of size calibration standards. Peaks from left to right represent blue dextran (MM 2 MDa), GSSG (MM ∼ 600 Da), GSH (MM ∼ 300 Da), and glycine (MM 75 Da). Stationary phase: Sephadex G-15 (31 × 1.0 cm I.D.); mobile phase: PBS-buffer (pH 7.4), flow rate: 1.0 mL/min; detector: ICP-AES at 193.091 nm; injection volume: 20 *μ*L.

As-specific detection was achieved with a Prodigy, high-dispersion, radial-view ICP-AES (Teledyne Leeman Labs, Hudson, NH, USA) by monitoring the As atomic emission line at 189.042 nm. Hyphenation of the LC system to the ICP-AES was accomplished by connecting the LC column exit to the concentric glass tube nebulizer with a polyethylene tube (38 cm, 0.13 mm I.D.). The plasma Ar gas-flow rate and the nebulizer gas pressure were 19 L/min and the radiofrequency power output was 1.3 kW. Time scans were performed using the time-resolved analysis mode (Salsa software version 3.0) with a data acquisition rate of one data point every 1.5 seconds. The data were exported and smoothed (bisquares weighting) using commercially available software (SigmaPlot 9.0). The chromatographic window of the packed Sephadex G-15 column was determined by injecting aqueous solutions of blue dextran (MM 2 MDa, *t_r_*~6 minutes), to define the exclusion volume (*V_0_*), and glycine (MM 75 Da, *t_r_*~15 minutes), to define the inclusion volume (*V_i_*). The column was size calibrated with aqueous solutions of GSSG (MM ∼ 600 Da, *t_r_*~10 minutes) and GSH (MM ∼ 300 Da, *t_r_*~13 minutes). All calibration experiments were performed with a PBS mobile phase (pH 7.4 at 25°C), while the C atomic emission line at (193.091 nm) was monitored (Figure 2). Previous studies have demonstrated that the pore size of Sephadex stationary phases does not change appreciably in the 4–37°C temperature range [22].

## 3. RESULTS AND DISCUSSION

**Figure 3:**
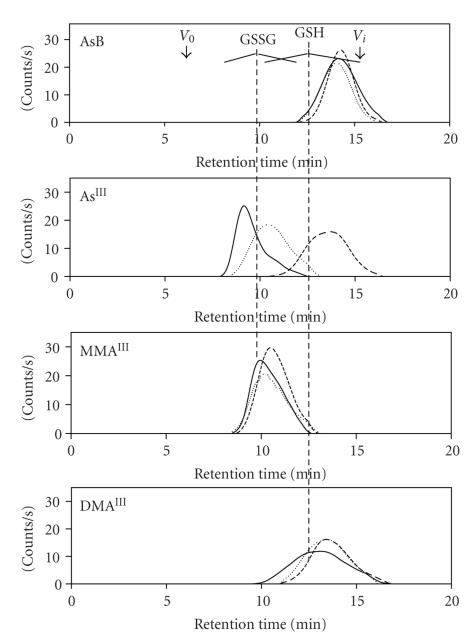
The superimposed chromatograms of AsB, As(III), MMA (III), and DMA(III) as a function of the SEC column temperature at 4°C (—), 25°C (- - -), or 37°C (– – –). Stationary phase: Sephadex G-15 (31 × 1.0 cm I.D.); mobile phase: PBS buffer containing 10 mM GSH adjusted to pH 7.4, flow rate: 1.0 mL/min; detector: ICP-AES at 189.042 nm; injection volume: 20 *μ*L (10 *μ*g As per compound).

Even though the mammalian metabolism of As(III) is not entirely understood, still As(SG)_3_, CH_3_As(SG)_2_, and (CH_3_)_2_AsSG likely play an important role [32, 43, 45, 47]. Despite this, only the stability of the As-S bonds in As(SG)_3_ have been investigated under conditions that resemble mammalian hepatocyte cytosol [22]. This was established using the “retention analysis method” which was originally developed to study the reversible oncolumn formation of drug-protein complexes by LC [58, 59]. Employing this approach, we studied the stability of trivalent As-(GS)_*x*_ complexes (where *x* = 1–3) by independently chromatographing As(III), MMA(III), and DMA(III) on an SEC column using GSH-containing PBS mobile phases (5 or 10 mM GSH, pH 7.4; Figure 3).

SEC was selected as the separation medium because previous studies into the reaction of the aforementioned trivalent arsenicals with GSH revealed the formation of As(SG)_3_ (MM ∼ 900 Da), CH_3_As(SG)_2_ (MM ∼ 600 Da), and (CH_3_)_2_AsSG (MM ∼ 300 Da) [20, 23, 48, 49, 60], which all differ in their hydrodynamic radii. Therefore, the ideal SEC stationary phase for the separation of the on-column formed complexes must have an appropriate fractionation window. The most suited SEC stationary phase was Sephadex G-15 since it offers an exclusion limit of <** **1500 Da [61]. Because of the particle size (120 *μ*m diameter) and particle size distribution (60–180 *μ*m) of this stationary phase [61], however, relatively broad chromatographic peaks are expected, as has been previously observed for As(SG)_3_ [22].

### 3.1. Temperature-dependent on-column formation/stability of trivalent As-(GS)_*x*_ complexes

To determine the influence of temperature on the on-column formation and stability of complexes that were formed between the injected trivalent As compounds and the mobile phase thiol (10 mM GSH), As(III), MMA(III), and DMA(III) were chromatographed on a Sephadex G-15 column at 4, 25, and 37°C. An additional arsenical, AsB (Figure 1(d)), that does not interact with GSH, was also chromatographed under these conditions as an internal standard. The observed retention behavior of As(III), MMA(III), DMA(III), and AsB is depicted in Figure 3.

In general, all four arsenicals eluted within the chromatographic window. As expected, AsB (MM ∼ 178 Da) eluted in the small-molecular-mass region between GSH (MM ∼ 300 Da) and glycine (MM 75 Da), irrespective of the column temperature (*t_r_*~14 minutes). In addition, the gradual reduction in peak width at baseline (*w_b_*) with an increase in temperature from 4 to 37°C can be rationalized by the faster rate of diffusion of AsB into and out of the pores. Based on the retention times of the peaks corresponding to As(III), MMA(III), and DMA(III) (*t_r_* range ∼9–13.5 minutes) compared to glycine (*V_i_*, *t_r_*~15 minutes) and the fact that a single chromatographic peak was obtained for each arsenical, they each must have eluted from the column in the form of their respective (GS)_*x*_-complexes at all investigated temperatures. This interpretation is further substantiated by the observation that As(III), MMA(III), and DMA(III) each eluted at ∼20 minutes, which is 5 minutes after the *V_i_*, when PBS-containing mobile phases without GSH were employed (data not shown). An unspecified chemical interaction between these trivalent arsenicals and the Sephadex G-15 matrix is most likely the cause of this behavior and has been previously observed for As(III) on a similar stationary phase material [22]. Presumably, the free hydroxyl groups of the Sephadex G-15 matrix (stemming from the dextran groups) interacted with the hydroxyl group(s) of the arsenicals via hydrogen bonding; hence, retarding their migration through the column. It is chemically improbable that the observed retention time changes between 4 and 37°C with the GSH-containing mobile phases were caused by the aforementioned unspecified chemical interaction because all injected arsenicals reacted first with GSH in the interstitial volume of the filter (∼80 *μ*L) prior to encountering the stationary phase pores.

### 3.2. Rationalization of the chemical structure of the on-column formed complexes

The elution order of the injected trivalent arsenicals at 4°C was As(III) (*t_r_*~9 minutes), MMA(III) (*t_r_*~10 minutes), and DMA(III) (*t_r_*~13 minutes), which implies that the hydrodynamic radii of the on-column formed trivalent As-(GS)_*x*_ complexes decreased in this order. Based on the known chemical affinity of As(III), MMA(III), and DMA(III) for GSH [22, 23, 62] (1)–(3), this order of elution strongly indicates that As(SG)_3_, CH_3_As(SG)_2_, and (CH_3_)_2_AsSG had formed on the column head. Evidence in favor of the on-column formation of As(SG)_3_ (MM ∼ 900 Da) comes from the observation that As(III) eluted between *V*_0_ and the MM 600 calibration standard (GSSG), which indicates that a complex with an MM between 1500 and 600 Da had formed (Figure 3). Similarly, CH_3_As(SG)_2_ and (CH_3_)_2_AsSG were most likely formed on the column head because the retention times corresponding to MMA(III) and DMA(III) were identical to those of GSSG (MM ∼600 Da) and GSH (MM ∼300 Da; Figure 3, vertical-dashed lines). These trivalent As-(GS)_*x*_ complexes could not be structurally characterized in the column effluent by electrospray ionization mass spectrometry (ESI-MS) because the temperature of the electrospray ion source chamber (120–365°C, Esquire 3000 ESI-quadrupole ion trap mass spectrometer) would have dissociated these thermally labile complexes (CH_3_As(SG)_2_ decomposes at 180°C, while (CH_3_)_2_AsSG decomposes at 100°C [23]) during the ionization process, prior to mass analysis. Moreover, as advised by the instrument supplier (Bruker Daltonics, Billerica, MA, USA), the salt concentration of mobile phase buffers that can be analyzed by ESI-MS must be below 10 mM (total salt in the utilized PBS buffer was ∼164 mM). Nevertheless, the alignment of the on-column formed complexes with the MM standards (Figure 3) in conjunction with the propensity of trivalent As compounds to react with soft thiol ligands in aqueous solution to form As(SG)_3_, CH_3_As(SG)_2_, and (CH_3_)_2_AsSG [20–24] strongly suggests that the latter species were formed on the column.

### 3.3. Retention behavior of As(III), MMA(III), and DMA(III) 

With regard to the retention behavior of As(III), the peak that was observed at 4°C showed considerable tailing, which suggests that As(SG)_3_ and an additional complex with a smaller hydrodynamic radius, possibly HOAs(SG)_2_, was formed on the column. An increase of the column temperature from 4 to 37°C resulted in a 4.6 minute retention shift of the As(III) peak toward the small-molecular-mass region (Figure 3), which can be interpreted in two different ways. Firstly, the alignment of this peak with GSSG at 25°C and with GSH at 37°C implies similar hydrodynamic radii, which indicates that HOAs(SG)_2_ and (HO)_2_AsSG were likely formed on the column under these conditions. Even though the peak corresponding to As(III) at 37°C did not perfectly align with the MM 300 calibration standard (GSH), it eluted closer to this standard than to glycine, which suggests that the As(III) was loosely bound to one GS-moiety. Alternatively, the three GS-moieties that are bound to As(III) at 4°C could undergo faster exchange upon increase of the temperature from 4 to 37°C (1). Nonetheless, these results strongly confirm the previously demonstrated lability of the As-S bonds in As(SG)_3_ [20, 22, 47–49].

The temperature-dependent retention behavior of MMA(III) and DMA(III) was significantly different from that of As(III) because the retention time of the peaks corresponding to MMA(III) and DMA(III) shifted only marginally (∼40 seconds for both versus 4.6 minutes for As(III)) upon an increase in the column temperature from 4 to 37°C (Figure 3). Hence the on-column formation of the corresponding trivalent As-(GS)_*x*_-complexes (where *x* = 1 or 2) was only minimally affected by the column temperature. This, in turn, implies that the As-S bonds in CH_3_As(SG)_2_ and (CH_3_)_2_AsSG are more stable than those in As(SG)_3_. Overall, these results, which were obtained under simulated physiological conditions, are in excellent accord with previous observations, which were conducted under nonphysiological conditions. In particular, these latter studies revealed that CH_3_As(SG)_2_ and (CH_3_)_2_AsSG could be synthesized in aqueous solution [23], whereas As(SG)_3_ could only be synthesized in alcoholic solutions (alcohol apparently stabilizes the hydrolytically labile As-S bonds) [20, 49].

The entire temperature-dependent retention behavior of As(III), MMA(III), DMA(III), and AsB was repeated with a 5 mM GSH-containing mobile phase (PBS, pH 7.4) on the same SEC column. These results were identical to those illustrated in Figure 3 (data not shown), which makes the observed comparative stability of the on-column formed complexes relevant to mammalian, protein-free, hepatocyte cytosol.

### 3.4. Biochemical ramifications of the obtained results

ATP-driven GS-X conjugate export pumps, which shuttle xenobiotic-GS-conjugates across phospholipid bilayer membranes via multidrug resistance proteins 1 and 2, are known to exist at the basolateral and apical hepatocyte membrane, respectively [42–45]. Therefore, our findings raise the possibility that the biomethylation of As(III) to MMA(III) and DMA(III) in the liver of mammals may have evolved simply to export As(III) into the bloodstream for subsequent urinary excretion via the kidney. This rather simplistic hypothesis could explain why DMA(III) has been detected in rat erythrocytes bound to hemoglobin [63] and why MMA(III) and DMA(III) have been detected in mammalian urine [12–16]. The exact mechanism by which these trivalent arsenicals are exported from the liver to the bloodstream, however, needs to be further investigated.

### 3.5. Influence of DMPS addition to the mobile phase on complex formation at 37°C

To substantiate that the retention shift of As(III) to a smaller retention time upon the addition of GSH to the mobile phase was caused by the on-column formation of As(SG)_3_, a mobile phase containing both GSH (5 mM) and DMPS (1 mM, Figure 1(f)), a chelating agent that forms a stronger complex with As(III) than GSH, was investigated. This resulted in the expected increase in the retention time of As(III) to *V_i_*(*t*_*r*_ ≈ 17 minutes), which is inaccord with previous findings [22]. In contrast, no As peaks for MMA(III) and DMA(III) were detected in the column effluent when a PBS mobile phase containing GSH (5 mM) and DMPS (1 mM) was employed. The cause of this reproducible behavior is not presently understood.

## 4. CONCLUSION

The SEC-based “retention analysis method” approach in conjunction with an As-specific detector was employed to study the comparative on-column formation of trivalent As-(GS)_*x*_-complexes. This was achieved by using mobile phases resembling the chemical composition of mammalian, protein-free hepatocyte cytosol (5 or 10 mM GSH, PBS, pH 7.4) at column temperatures of 4, 25, or 37°C. The separate injections of As(III), MMA(III), and DMA(III) and their observed retention behavior provided evidence for the on-column formation of more stable As-S bonds in CH_3_As(SG)_2_ and (CH_3_)_2_AsSG than in As(SG)_3_. These findings imply that the stability of As-S bonds could be critically involved in the disposition/excretion of methylated trivalent As compounds in mammals. Future investigations should be aimed at identifying whether CH_3_As(SG)_2_ and (CH_3_)_2_AsSG are in fact translocated across the hepatocyte membrane into the bloodstream *in vivo.*
